# Formation and compaction of sub-10 nm transfer films in metal–ceramic fretting

**DOI:** 10.3762/bjnano.17.83

**Published:** 2026-08-28

**Authors:** Tunç Çiftçi, Ozan Şahin, Thibault Roch, Bart Weber

**Affiliations:** 1 Advanced Research Center for Nanolithography (ARCNL), Science Park 106, 1098 XG Amsterdam, The Netherlands; 2 Van der Waals-Zeeman Institute, Institute of Physics, University of Amsterdam, Science Park 904, 1098 XH Amsterdam, The Netherlands

**Keywords:** adhesive wear, atomic force microscopy, fretting, metal–ceramic interface, transfer film

## Abstract

Nanoscale transfer in metal–ceramic fretting originates at microcontacts but has remained experimentally unresolved. Here, we use an atomic force microscopy-based topographical difference method to visualize the formation of sub-10 nm transfer films at a sapphire–chromium interface subjected to fretting. Transfer proceeds via adhesive wear of chromium at micrometer-scale contacts, followed by plastic compaction of the chromium debris into a conformal ≈5 nm transfer film. Excess debris is expelled from the circular contact and accumulates in a curvature-defined peripheral ring around the contact. These results connect microcontact fracture, debris evolution, and nanoscale transfer-film formation in fretting contacts.

## Introduction

Precision interfaces subjected to cyclic tangential forces are designed to avoid gross sliding, yet they can undergo progressive nanoscale material transfer [1–3]. Even a few nanometers of redistributed material can alter the interfacial topography, leading not only to measurable height offsets [1] but also to changes in local roughness that affect friction and adhesion response. In semiconductor lithography, such transfer at metal–ceramic contacts [4–6] can degrade overlay and focus stability. However, the physical processes by which microcontacts transition from deformation to material transfer under partial slip [7] remain poorly understood.

The onset of material transfer under partial slip is governed by the local contact mechanics of rough interfaces [8–9]. Classical Hertzian theory [10] describes the stress distribution in elastic sphere-on-flat contacts, while the partial-slip solutions of Cattaneo [11–12], Mindlin [13], and Johnson [14–15] show that tangential loading produces a central stick region surrounded by a slipping annulus. In real, rough contacts, however, the interface is composed of discrete microcontacts, each experiencing different normal and tangential stresses [16]. As a result, local stress ratios can approach or exceed thresholds for slip or fracture even when the nominal interface remains below the macroscopic sliding limit [17–22].

Classical contact theories assume smooth bodies [10,13], whereas real interfaces are rough and composed of interacting asperities across multiple length scales [23]. Multiscale contact mechanics frameworks, such as that of Persson [24–27], describe how load is distributed over a hierarchy of microcontacts and how the real contact area evolves with applied pressure. Crucially, this stress redistribution leads to highly heterogeneous local conditions, with some asperities experiencing elevated pressures and shear stresses [25,27].

Building on multiscale contact mechanics, recent multiasperity friction experiments have shown that individual microcontacts within a nominal interface can simultaneously occupy subcritical, partial-slip, and critically loaded states [28]. This heterogeneity implies that even when the macroscopic interface remains below the sliding threshold, certain asperities may experience stresses sufficient to induce slip or material detachment. Early tribological studies by Miyoshi and co-workers [5–6] demonstrated that adhesive interactions in metal–ceramic contacts can promote local plastic shearing and material transfer once partial slip develops. However, these macroscopic observations did not resolve how debris nucleates and evolves at the nanoscale [29] within a realistic multiasperity contact.

Adhesive wear can be understood in terms of a critical length scale that separates plastic smoothing from fracture-mediated debris formation. Adhesive wear refers to the broader wear process initiated by the formation and shearing of adhesive junctions between contacting asperities. Fracture-mediated debris formation denotes the specific detachment mechanism that occurs when such junctions exceed the critical length scale and fracture rather than deform plastically. Aghababaei and Molinari showed that contact patches smaller than a material-dependent threshold deform plastically [30], whereas larger junctions fracture and generate wear particles [31]. Under reciprocated sliding, such debris can spread, compact, and evolve into extended third bodies or transfer films [32–33]. However, how this transition from microcontact growth to debris formation and film evolution manifests within realistic multiasperity contacts [34] operating under partial slip remains experimentally unresolved.

Experimentally, fretting-induced transfer has primarily been characterized at the microscale using optical microscopy, profilometry, SEM, EDX, and TEM. These techniques reveal wear scars, debris accumulation, and mature tribofilms, but are typically applied after material redistribution has reached micrometer dimensions [6,35–38]. As a result, the transition from asperity-scale deformation to debris nucleation and nanoscale transfer-film formation remains unresolved. Although atomic force microscopy (AFM) offers sub-nanometer sensitivity to topography, adhesion, and dissipation [39–45], it has only rarely been applied to visualize the onset of transfer across a realistic multiasperity fretting interface [46].

In this work, we combine controlled partial-slip fretting with nanoscale-resolved AFM topography difference mapping to resolve the onset of transfer-film formation at a multiasperity sapphire–chromium interface. We directly visualize the nucleation of a sub-10 nm conformal transfer film and correlate it with micrometer-scale crater formation on the chromium surface. By integrating contact mechanics simulations with critical length scale analysis, we examine when microcontacts transition from plastic deformation to fracture-mediated debris generation. This approach establishes a mechanistic link between asperity-scale fracture, debris evolution, and nanoscale transfer-film formation in fretting contacts.

## Experimental

**Figure 1:**
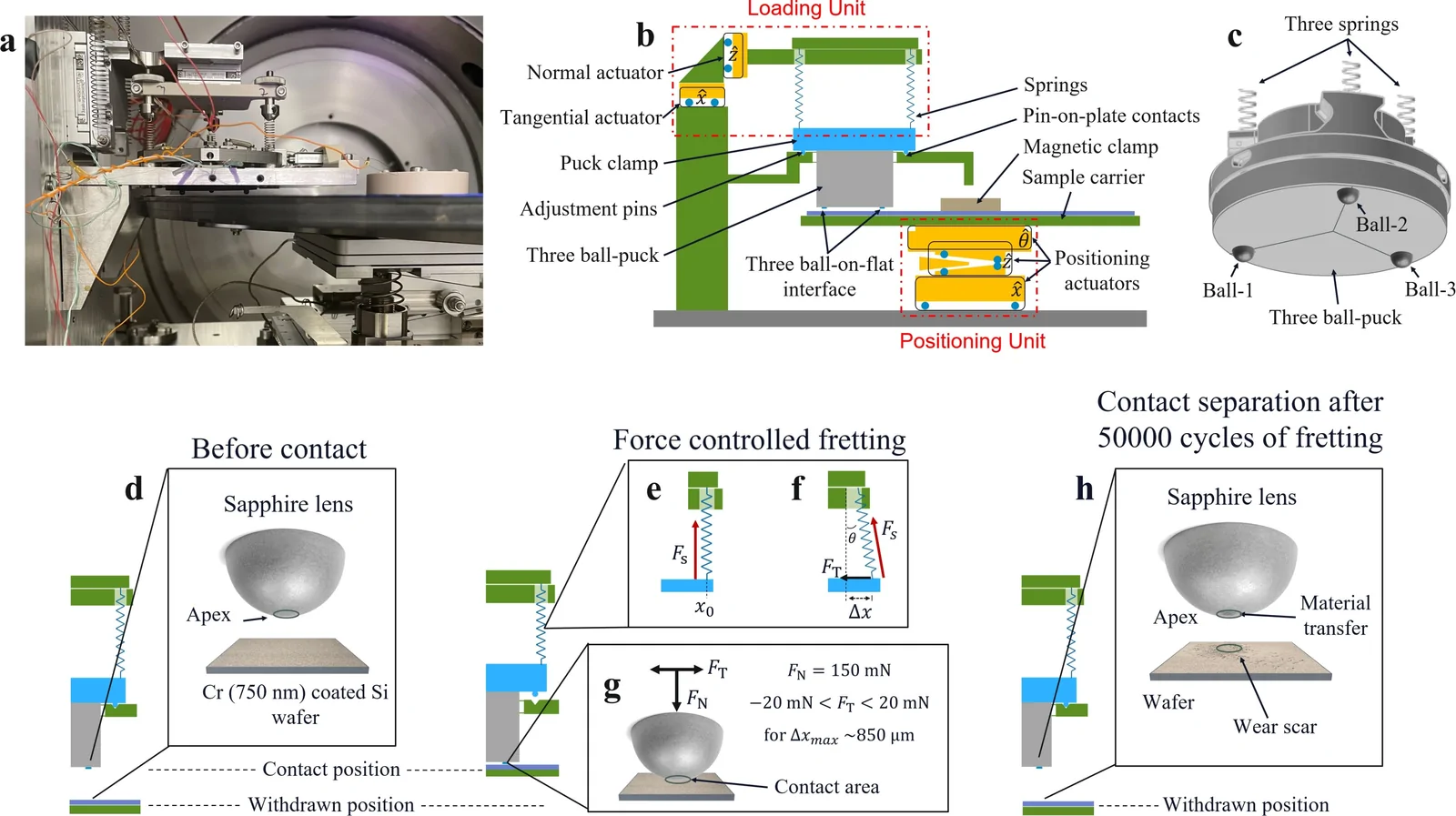
(a) Photograph of the customized tribometer installed inside a vacuum chamber. (b) Two-dimensional schematic of the tribometer reproduced from [21] (© 2023 J. Du et al., distributed under the terms of the https://creativecommons.org/licenses/by/4.0/ Creative Commons Attribution 4.0 International License). The setup consists of a loading unit with normal and tangential actuators coupled to a three-ball puck through springs, and a positioning unit providing lateral (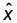), normal (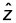), and rotational (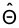) motion of the wafer. (c) Three-ball puck assembly (280 g). (d–h) Fretting protocol: (d) aligned sapphire lens (*R* = 1.59 mm) and Cr-coated Si wafer (Cr thickness 750 nm) before contact; (e) application of the normal load *F*_N_ through spring loading; (f) oscillatory lateral displacement Δ*x*_max_ generating tangential force *F*_T_; (g) imposed loading conditions (*F*_N_ = 150 mN, −20 *< F*_T_ *<* 20 mN, Δ*x*_max_ ≈ ±850 μm); (h) chromium transfer film on sapphire and corresponding wear scar on the Cr-coated wafer after 50,000 fretting cycles.

To ensure optimally aligned and force-controlled contact and fretting, we employed a customized three-ball-on-flat tribometer (Figure 1a) [21] located inside a clean room (ISO14644-Part 1, Class 6). The instrument consists of (Figure 1b): (1) a loading unit containing normal and tangential actuators coupled to the three-ball puck through three springs (TEVEMA T40730A, spring constant *k* = 3.64 N·mm^−1^, free length *L*_0_ = 17.7 mm, maximum extension S*_n_* = 55.93 mm, and (2) a positioning unit providing lateral (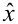), normal (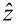), and rotational (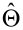) motion for precise positioning of the wafer substrate, which is fixed with a magnetic clamp onto the sample carrier beneath the three-ball puck (Figure 1c).

The three springs mechanically couple the loading unit to the puck holder, enabling control over the three-ball-on-wafer normal and tangential force. Application of an oscillating tangential force can thus lead to fretting conditions when this tangential force remains below the friction threshold of the system. During fretting, the interface undergoes only partial slip rather than gross sliding [37].

As the ceramic counter bodies, we used three polished sapphire half-ball lenses (Edmund Optics; diameter 3.18 mm). Sapphire was chosen because it is a hard, chemically inert ceramic representative of the materials used in precision positioning interfaces. The metallic counterpart was a chromium-coated silicon wafer comprising a 750 nm thick Cr film on a p-type Si substrate (Siegert Wafer, ⟨100⟩, boron-doped, 500–525 μm thick, 1–10 Ω·cm) coated using magnetron sputtering physical vapor deposition. Chromium films are widely employed as adhesion, reference, and clamping-contact layers in semiconductor tooling, making this sapphire–Cr pair a representative model system for industrial metal–ceramic fretting interfaces.

Before loading, the puck assembly rested on three adjustment pins seated in pin-on-plate contacts (Figure 1d), which were previously aligned with the sample on the positioning unit. Using the positioning unit, the wafer was positioned beneath the sapphire lenses. The puck assembly (Figure 1d) weighs 280 g, resulting in a downward gravitational force of approximately 2.75 N. To reduce the puck-on-wafer contact force, the three springs apply tension, such that the puck is resting on the pin-on-plate with a total normal force of 450 mN (150 mN per contact). In other words, the springs compensate part of the gravitational force on the puck. As the wafer was subsequently lifted toward the puck, making contact with the sapphire lenses, the adjustment pins disengaged and 150 mN normal force was applied to each sapphire-on-wafer contact (Figure 1e,g).

To ensure that the sapphire–wafer interface operated in the pre-sliding regime, we applied a sinusoidal tangential displacement at ≈0.2 Hz using the tangential actuator connected to the upper ends of the springs. The actuator motion imposed a lateral displacement amplitude of Δ*x*_max_ ≈ ±850 μm, generating a corresponding tangential force of −20 *< F*_T_ *<* 20 mN on each sapphire ball-on-wafer contact (Figure 1f,g). Together with the applied normal load of *F*_N_ = 150 mN, this loading ratio maintains the local tangential-to-normal stress ratio (μ = *F*_T_/*F*_N_ ≈ 0.13) below the reported friction threshold values for sapphire–metal interfaces (μ ≈ 0.5) [47–48], thereby initiating controlled partial slip without inducing gross sliding of the microcontacts within the three-ball-on-flat interface. We subjected the interface to 50,000 fretting cycles under these loading conditions. This cycle count was selected as a practical compromise between generating sufficient material transfer for detailed AFM characterization and maintaining experimentally feasible measurement times.

Surface characterization before and after fretting was performed using a Bruker Dimension Icon AFM operated in PeakForce QNM mode with a diamond probe (Adama Innovations AD-2.8-AS; spring constant 2.8 N·m^−1^, resonance frequency 65 kHz, apex radius 10 ± 5 nm). Topography and adhesion maps were simultaneously acquired to assess transfer-film development and changes in interfacial properties. Detecting changes in topography at the nanometer scale is challenging. Therefore, we complemented the topography measurements with local adhesion measurements, as adhesion is a sensitive indicator of local chemical and mechanical heterogeneity [43,45]. The smoothness of both the sapphire and Cr-coated wafer surfaces ensures stable high-resolution operation, while the robust diamond tip resists wear. The low spring constant offers enhanced force sensitivity and tolerates topographic variation over larger scanned regions at the ball apex.

To quantitatively examine how roughness evolves across spatial scales, we computed the root-mean-square (RMS) roughness as a function of scan window size using a sliding-window approach [49–50]. This analysis was applied to AFM topographs acquired from both the sapphire and chromium surfaces.

To gain further insight into the interfacial contact behavior and enable predictive modeling, we used Tamaas, which implements an FFT-accelerated boundary and volume integral method [51–52] for solving rough elasto-plastic contact problems (see Supporting Information File 1 for details). By conducting elasto-plastic contact computations of the sapphire–chromium interface, we resolved the contact geometry, local pressure distribution, displacement fields, interfacial gap evolution, and plastic strains under applied loads. AFM topographs of both the sapphire apex and the chromium-coated wafer recorded prior to fretting served as input for these calculations, enabling a realistic simulation of the elastic and plastic contact response at the interface.

## Results

**Figure 2:**
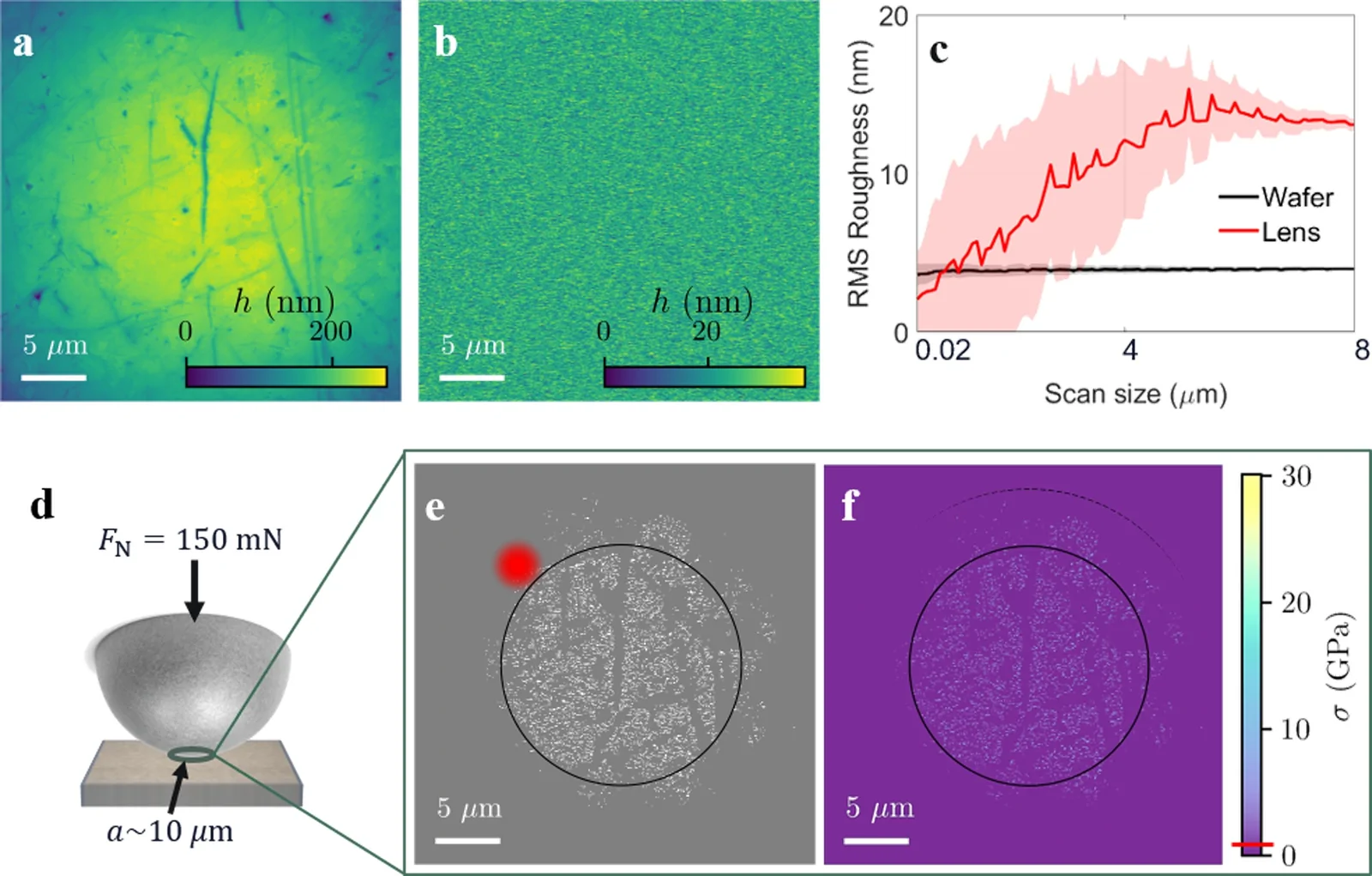
AFM topography acquired from (a) the sapphire apex and (b) the chromium film. (c) Scale-dependent root-mean-square (RMS) roughness evaluated over sliding square windows [49–50] of increasing scan size for the counterparts before fretting. The lines denote the mean RMS value over all possible window positions for a given size, and the shaded areas represent the corresponding standard deviation. (d) Schematic of the sapphire-on-chromium contact under the applied normal load *F*_N_ = 150 mN. Contact simulations [51–52] based on the measured topographies yield (e) a binary contact map and (f) the local contact pressure distribution. The black circles indicate the estimated Hertzian contact area (with radius *a* ≈ 10 μm). The red disk in (e) provides a visual reference for the estimated critical length scale d^*^; several micrometer-scale contact clusters are comparable in lateral size and are therefore candidates for fracture-mediated debris formation, as discussed below. The red marker on the color bar in (f) denotes 1 GPa, above which local plastic deformation of the chromium film is expected.

Figure 2a and Figure 2b show the AFM scans acquired on the sapphire sphere and the chromium surface, respectively. Figure 2c displays the scale-dependent RMS roughness, evaluated using sliding square windows of increasing size [49–50], extracted from the AFM images shown in Figure 2a and Figure 2b. The chromium surface exhibits consistently low roughness values around 4 nm, indicating high uniformity. In contrast, the RMS roughness of the sapphire lens increases with window size and stabilizes beyond 8 × 8 μm^2^. The error ranges indicated by shaded areas in Figure 2c represent the spatial variability across the scanned region, demonstrating that roughness is highly location- and scale-dependent, especially for inhomogeneous surfaces like the polished sapphire apex.

Microcontact size and local contact pressure are key parameters governing material transfer during fretting. To obtain predictive insight, we combined classical Hertzian contact mechanics [10,15] with Tamaas-based [51–52] elasto-plastic contact calculations. Figure 2d illustrates the classical Hertzian approach for a sapphire lens contacting a chromium-coated surface. With sapphire and chromium Young’s moduli of 335 GPa [53] and 279 GPa [54–55], respectively, we computed the reduced modulus E^*^ ≈ 161 GPa. At a contact force of 150 mN, and with a lens diameter of 3.18 mm, Hertz theory yields a contact radius *a* ≈ 10 μm, a maximum contact pressure *p*_max_ ≈ 693 MPa, and an average contact pressure *p*_avg_ ≈ 462 MPa.

The AFM topographies shown in Figure 2a and Figure 2b served as input for the elasto-plastic numerical calculations performed with Tamaas [51] (see Supporting Information File 1 for details). The contact map in Figure 2e reveals that the interface is composed of multiple discrete microcontacts, mostly within the calculated Hertzian contact. Several contact clusters reach micrometer-scale lateral dimensions comparable to the red disk, which provides a visual reference for the estimated critical length scale for adhesive wear. These clusters are therefore candidates for fracture-mediated debris formation (see Discussion section). In addition, Figure 2f shows that many contact regions experience local pressures exceeding 1 GPa, as indicated by the red marker on the color bar, suggesting that plastic deformation of the chromium film may occur during the experiment, thereby justifying the use of an elasto-plastic rather than a purely elastic contact calculation. The real contact area in the elastic-plastic computation is roughly 30% larger than in a purely elastic computation. To quantify material transfer experimentally, we performed a comparative analysis of AFM topographies acquired before and after fretting.

**Figure 3:**
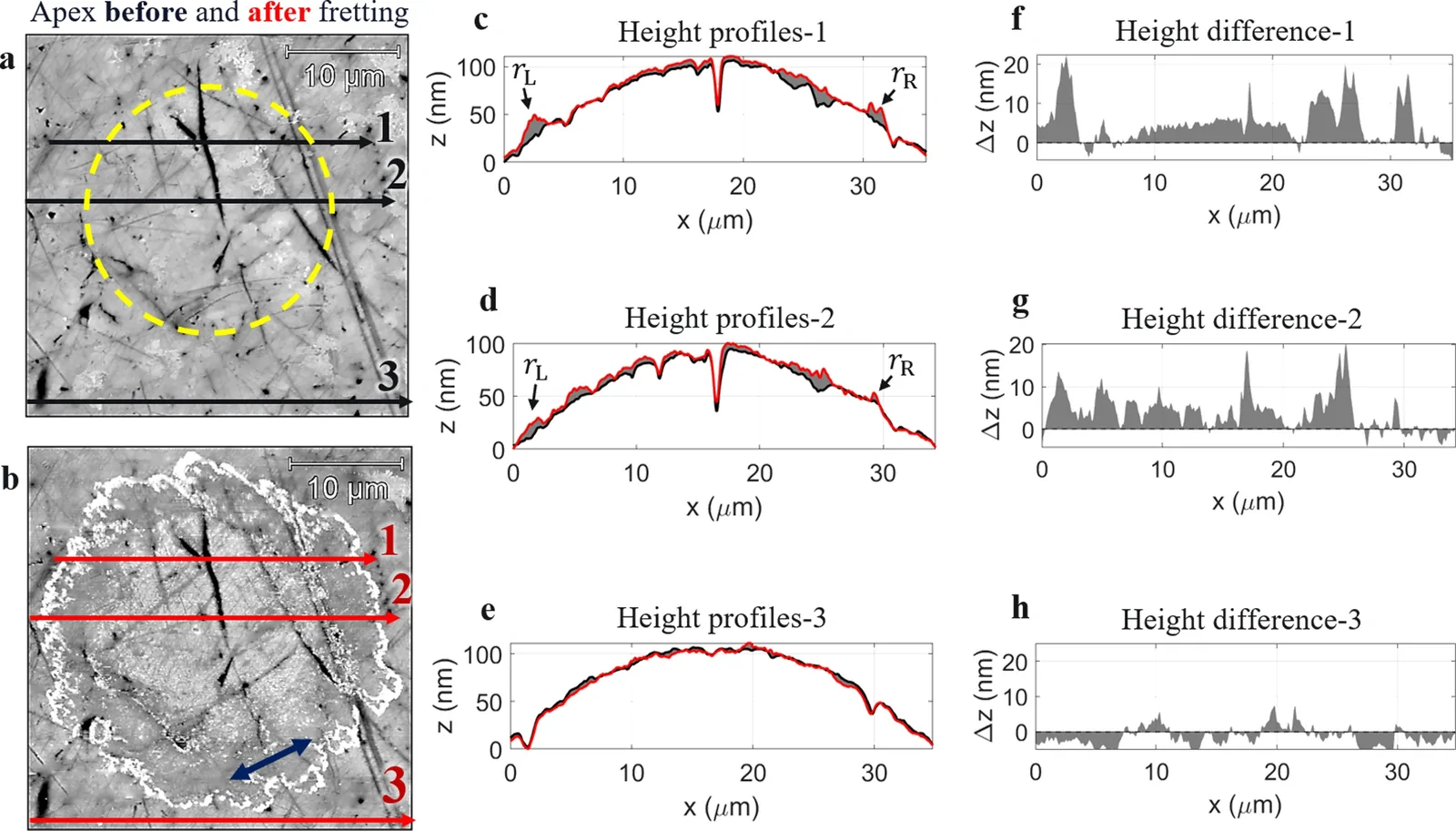
AFM height maps of the sapphire apex (a) before and (b) after fretting from a 34 μm × 34 μm scan area (512 × 512 pixels). The yellow dashed circle indicates the Hertzian contact area, and the dark blue arrow indicates the fretting direction. A nanometric transfer film forms within the contact region in (b), within the predicted Hertzian contact radius (≈10 μm), while the peripheral ring reveals a thicker profile as indicated by *r*_L_ and *r*_R_. (c,d) The profiles show that trenches visible before and after fretting are partially filled by transferred material. (c–e) Height profiles represent the black and red arrows from (a) and (b). Each displayed height profile represents the average of 128 neighboring AFM scan lines, corresponding to a bandwidth of approximately 8.5 μm. (f–h) Height difference plots taken from the gray-shaded areas between the black and the red profiles in (c–e).

Using a topographical difference approach, we extracted co-located cross-sectional height profiles to assess the material transfer and the spatial extent of surface modification. Each displayed height profile represents the average of 128 neighboring AFM scan lines, corresponding to a bandwidth of approximately 8.5 μm. Figure 3a and Figure 3b show AFM height maps of the sapphire apex measured before and after fretting. The sapphire surface initially exhibits shallow trenches and nanoscale polishing marks. After fretting, two distinct transfer regions appear: The first is a thin, conformal transfer film covering the predicted Hertzian contact area, and the second is a surrounding narrow outer ring consisting of relatively larger debris particles. Trenches visible in both images are partially filled with transferred material from the chromium surface.

Height profiles in Figure 3c–e extracted along the arrows in Figure 3a and Figure 3b quantitatively compare the topographic evolution. Height and difference profiles reveal a median height increase of approximately 5 nm within the Hertzian contact area (Figure 3f and Figure 3g). The height profiles measured after fretting exhibit two protrusions from the peripheral ring, denoted as *r*_L_ and *r*_R_, with peak heights of 15–20 nm. The peripheral ring of transferred debris is approximately 4 μm outside the Hertzian contact. Furthermore, the two broader grooves on the sapphire surface become progressively filled by transferred debris, as indicated by the local height increase in the difference profiles (Figure 3c,d,f,g). Outside the wear scar, only a minimal difference between before and after topography measurement is observed (≈0.2 nm), confirming the stability of the tip and repeatability of the topographic measurement.

**Figure 4:**
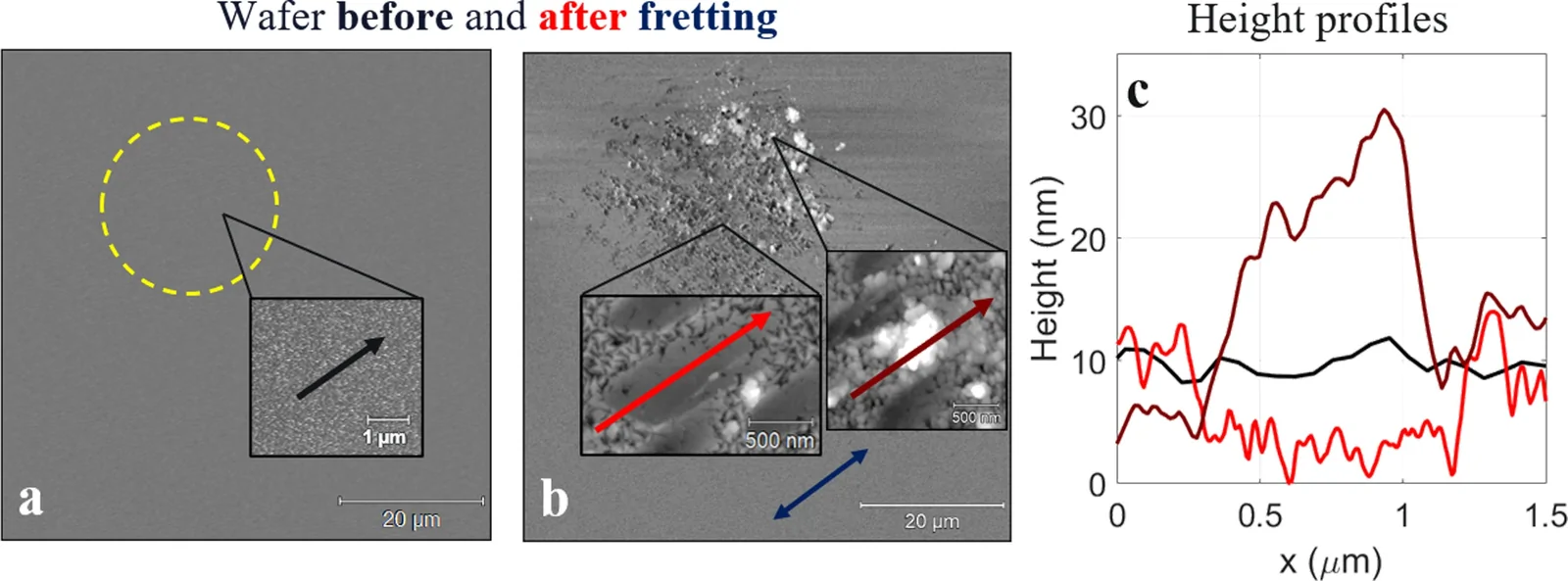
AFM topography images of the chromium-coated wafer (a) before and (b) after fretting, acquired over a 68 μm × 68 μm area (1024 × 1024 pixels). The post-fretting image exhibits a well-defined wear scar that overlaps with the Hertzian contact area, indicated by the yellow dashed circle. The insets highlight (a) the pristine chromium surface covered by a native oxide and (b) local fretting-induced features, including craters in the center and debris accumulation at the periphery. The dark blue arrow denotes the fretting direction. (c) Height profiles reveal the surface modulation induced by fretting. Each displayed height profile represents the average of 32 neighboring AFM scan lines, corresponding to a bandwidth of approximately 250 nm.

Figure 4a and Figure 4b show AFM height maps of the chromium-coated wafer before and after fretting, providing direct evidence of fretting-induced surface restructuring and material displacement. Prior to fretting, the chromium surface exhibits a uniform granular texture with minimal height variation. After fretting, a well-defined wear scar develops within and around the Hertzian contact region, with a lateral extent that closely matches the material transfer zone observed on the sapphire surface (Figure 3b).

The insets in Figure 4b highlight local craters formed near the center of the wear scar and debris accumulation at the periphery. Each displayed height profile in Figure 4c, extracted along the marked lines in Figure 4a,b, represents the average of 32 neighboring AFM scan lines, corresponding to a band width of approximately 250 nm. The craters are elongated along the fretting direction and are surrounded by flake-like surface features, indicating a shear-driven detachment process. It is important to emphasize the aspect ratios of the craters and debris particles: Both the craters and the debris particles are extended in the contact plane, in some cases beyond a micrometer, but very shallow (tens of nanometers in depth/height; see Figure 4).

**Figure 5:**
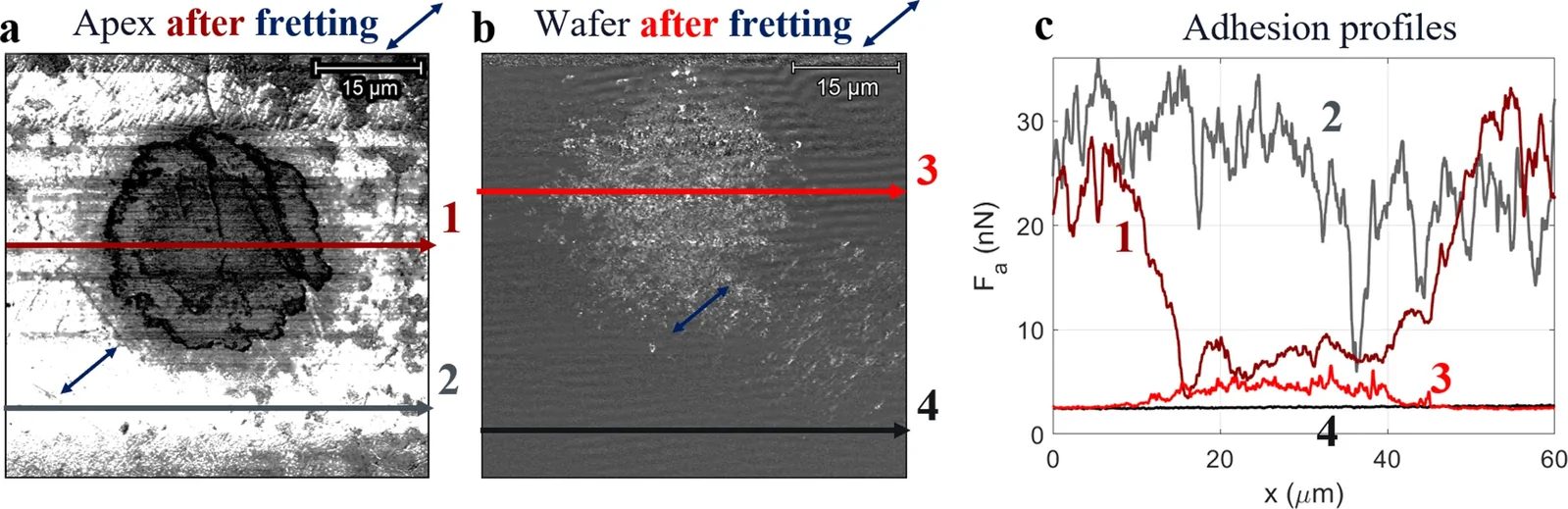
Adhesion maps of (a) the sapphire apex and (b) the wear scar on the chromium surface after fretting measured in PeakForce QNM mode using the same AFM tip (Adama Innovations, AD-2.8-AS). The dark blue arrow indicates the fretting direction. (c) Adhesion profiles extracted along the corresponding marked positions (in a and b) represent the average of 128 neighboring scan lines. The dark red profile along the transferred material at the ball apex exhibits lower adhesion compared to the dark gray profile from surrounding pristine sapphire regions. The red profile along the wear scar on the chromium-coated wafer shows higher adhesion compared to the black profile from surrounding flat chromium film. The contrast between reduced adhesion on the sapphire and enhanced adhesion on the chromium confirms asymmetric interfacial evolution during material transfer, correlating with the topographic profiles shown in Figure 3 and Figure 4.

The adhesion measurements highlight local contrast in adhesion likely caused by material transfer. On the sapphire apex (Figure 5a), the region on the surface at which material transfer was identified (see Figure 3) shows significantly lower adhesion than pristine sapphire measured outside the wear scar, indicating a distinct surface composition within the transfer film. On the chromium-coated wafer (Figure 5b), the wear scar exhibits a higher adhesion signal compared to the surrounding smooth surface. The observed adhesion contrast between sapphire and chromium surfaces demonstrates complementary changes at both sides of the contact.

Note that we used the same AFM tip for the before and after comparison measurements under identical acquisition conditions to minimize probe-induced artifacts. The corresponding height-difference profile outside the fretting contact exhibited a residual variation of only approximately 0.2 nm (Figure 3e,h), while the post-fretting adhesion contrast was spatially confined to the transfer-film and wear-scar regions (Figure 5). These observations indicate that no measurable probe blunting or contamination affected the reported topographic and adhesion measurements. Nevertheless, atomic-scale wear of the diamond apex below the sensitivity of these controls cannot be excluded [56].

## Discussion

### From microcontacts to debris generation

Our results demonstrate that the sapphire-on-chromium interface forms sufficiently strong microcontacts to initiate material transfer from the chromium film onto the sapphire surface during fretting. We hypothesize that, as fretting proceeds, the sapphire-on-chromium microcontacts (Figure 2) generate sufficient stress to induce adhesive wear of the chromium film, resulting in chromium debris formation. The observations of craters and debris particles on the chromium surface (Figure 4) are consistent with this interpretation.

To evaluate whether the simulated microcontacts (Figure 2e,f) can generate chromium debris through adhesive wear, we apply the critical length scale model developed by Aghababaei and Molinari [31]. This model defines a threshold lateral dimension d^*^ of contact patches, above which crack propagation becomes energetically favorable, initiating debris formation via brittle fracture [57]. The critical size is given by




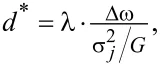




where Δ*w* is the surface energy of the material to be fractured, σ*_j_* is the shear strength of the contact patch, *G* is the shear modulus of the softer material, and λ is a dimensionless geometric constant. For chromium [5], we adopt Δ*w* = 2.25 ± 0.15 J·m^−2^ and *G* = 110 ± 10 GPa [58–59]. We estimate the contact patch shear strength as σ*_j_* = μ*p*, where μ ≈ 0.5 is a representative friction coefficient for sapphire–chromium interfaces under fretting conditions [47–48], and ≈1 GPa is the characteristic normal stress acting within load-bearing microcontacts (Figure 2e,f).

Since both the craters and detached debris in Figure 4 are laterally extended in the contact plane but very shallow (tens of nanometers in depth/height), we treat the wear mechanism as plate-like rather than spherical. Following Aghababaei and Molinari [31], we therefore take the geometric factor λ in the range from 1 to 8/π, where 8/π ≈ 2.55 corresponds to an idealized 2D circular particle. Using σ*_j_* = 500 MPa, Δ*w* = 2.10–2.40 J·m^−2^, and *G* = 100–120 GPa, we conclude that d^*^ should be in the range of 800 nm to 5 μm.

This analysis indicates that brittle debris formation becomes feasible once sapphire–chromium contacts grow or coalesce into micrometer-scale contact patches. Based on the contact calculations in Figure 2d,f, the load-bearing contact patches span sub-micrometer to micrometer lateral dimensions, with clustered contact patches frequently exceeding 1 μm and reaching several micrometers. Importantly, experimental evidence supports the existence of such large contact patches: Figure 4b shows micrometer-scale wear-induced features distributed within the nominal Hertzian contact region (radius ≈10 μm). Moreover, the insets in Figure 4b and the corresponding height profiles in Figure 4c reveal crater-like features that reach lateral dimensions of up to ≈1 μm.

**Figure 6:**
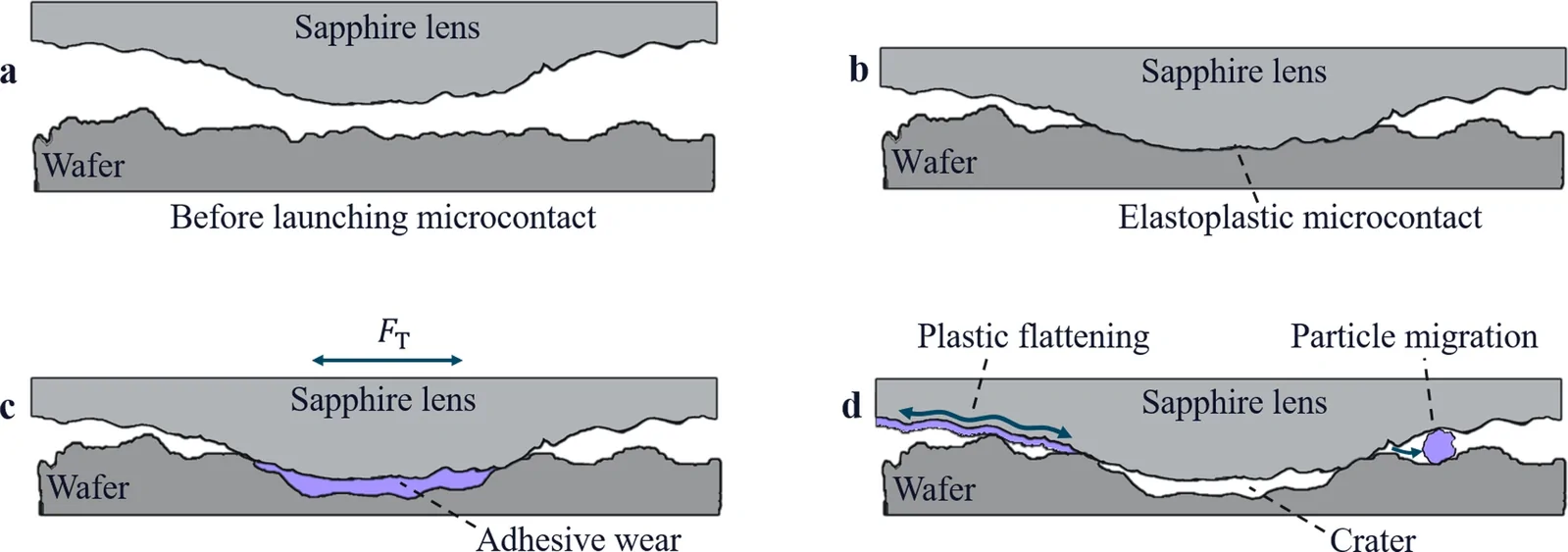
Schematic illustration of the local evolution of a representative microcontact within the multiasperity sapphire–chromium interface: (a, b) microcontact formation, (c) fretting-induced adhesive wear and debris generation, and (d) subsequent compaction and redistribution of the debris. Similar processes may occur simultaneously at multiple microcontacts. The schematic does not imply complete removal of debris from the interface. Rather, only a fraction of the debris remains trapped and is compacted into the transfer film, whereas excess debris is redistributed between neighboring microcontacts, into sufficiently deep surface features on the sapphire, or toward the contact periphery (see Figure 3). Residual debris and craters also remain within the wear scar on the chromium surface. The craters typically have lateral dimensions of the order of 1 μm and depths of the order of 10 nm (see Figure 4).

Overall, Figure 4b and Figure 4c place most fretting-generated craters and detached features directly within the predicted critical regime for brittle fracture. Together, these observations suggest that fretting-induced adhesive wear in our system proceeds via the nucleation of micrometer-scale fractured debris, as schematically illustrated in Figure 6, and motivate a closer examination of the resulting crater morphology and transfer layers discussed below.

### How debris accumulates: craters and transfer layers

Craters in Figure 4b are elongated and aligned with the fretting direction within the Hertzian contact zone, indicating that damage accumulation is governed by the imposed cyclic shear rather than purely normal loading. This directional morphology supports the adhesive wear scenario depicted in Figure 6c, where repeated tangential loading promotes growth in contact patches and subsequent detachment events. Following detachment, the cratered regions remain exposed to continued fretting, which may enable further fragmentation and redistribution of chromium fragments within the contact.

The chromium debris generated during fretting is likely plastically compacted under repeated loading, forming conformal contact with the sapphire surface and thereby contributing to the formation of the thin (≈5 nm) transfer film observed experimentally. Such transfer-film formation, in which detached material spreads and compacts within the contact, is reminiscent of recent mechanistic descriptions of metallic wear governed by transfer layer formation and interfacial cracking [33].

Because the present measurements are ex situ, the transient morphology between debris detachment and compaction is not directly resolved. Nevertheless, the coexistence of craters and debris on the chromium surface, the compacted transfer film on the sapphire, and the peripheral debris ring, together with the critical-length-scale analysis, is consistent with successive debris generation, redistribution, and compaction. Whether the approximately 5 nm thick conformal film develops gradually toward a steady-state thickness or continues to evolve under prolonged fretting, for example after renewed contact with fresh chromium, remains an interesting topic for future investigation.

### The peripheral ring: a geometric escape zone

AFM measurements of the sapphire apex reveal two distinct topographic features after fretting, namely, a conformal transfer film within the Hertzian contact and a peripheral debris ring located just outside the contact (Figure 3). The transfer film likely forms through plastic compaction of fine chromium debris trapped within the contact (Figure 6d). In contrast, the formation of the peripheral ring requires debris particles to migrate radially outward beyond the Hertzian contact area.

**Figure 7:**
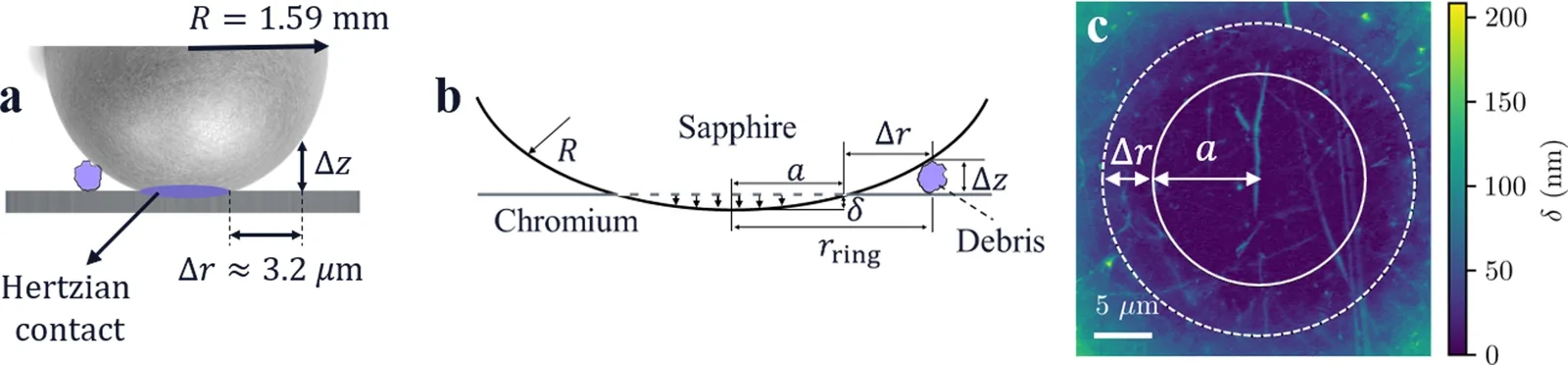
Formation of the peripheral debris ring during sapphire–chromium contact. (a) Schematic illustration of the Hertzian contact between a sapphire lens with radius *R* = 1.59 mm and a chromium-coated wafer. (b) Cross-sectional representation of the contact geometry showing the radial position *r*_ring_ where the gap between the sapphire surface and the chromium film Δ*z* should be at least equal to the debris particle height. The Hertzian contact radius is *a*, and the elastic indentation depth is δ. A peripheral debris ring forms at a radial offset Δ*r* from the contact edge. (c) Interfacial gap map obtained from elasto-plastic contact simulations based on the Tamaas library [51–52].

For a particle to escape the contact, it must reach a radial position Δ*r* where the vertical gap between the sapphire lens and the wafer Δ*z* equals or exceeds the particle height, as illustrated schematically in Figure 7a,b. Assuming a representative debris particle height of *z* ≈ 25 nm (Figure 4c) and a sapphire lens radius of *R* = 1.59 mm, we estimate that the gap equals the particle height at a radial distance *r*_ring_ *>* 13.5 μm from the contact center, so Δ*r >* 3 μm from the Hertzian contact edge (see Supporting Information File 1 for details).

This estimate agrees well with the experimentally observed debris ring position shown in Figure 3. It also coincides with the predicted peripheral ring (dashed circle) in the simulated interfacial gap map in Figure 7c. Together, these results support a curvature-limited redistribution mechanism in which debris particles escape the contact and accumulate at radial positions where the geometry provides sufficient vertical clearance.

## Conclusion

We present sapphire-on-chromium fretting experiments combined with detailed atomic force microscopy analysis of the resulting wear scars. Using a topography difference method, we identify the formation of a conformal chromium-based transfer film of approximately 5 nm thickness on the sapphire surface after fretting. Adhesion measurements reveal a clear contrast between the transfer film and surrounding pristine sapphire regions, confirming the presence of a mechanically distinct interfacial layer.

Our observations are consistent with a two-step mechanistic interpretation: (1) Chromium fragments detach from the coating by fracture during adhesive wear at micrometer-scale contact patches, and (2) the resulting debris particles are plastically compacted under continued cyclic shear and adhere to the sapphire surface, forming a thin conformal layer within the Hertzian contact region.

Debris generated near the edge of the contact is sheared radially outward. These larger particles accumulate in a ring-shaped region where the local interfacial gap, set by the lens curvature, becomes comparable to the particle height and can accommodate their presence in the form of a peripheral ring just outside the contact area.

Together, these results show that nanoscale transfer in partial-slip fretting proceeds through debris formation, in-contact compaction into a conformal film, and curvature-controlled expulsion of excess material. Although demonstrated here for the sapphire–chromium interface, the present approach provides a framework for connecting multiasperity contact mechanics with critical-length-scale theory in realistic tribological systems. Given the appropriate material properties and contact conditions, the framework can, in principle, be applied to predict whether microcontacts are expected to reach the critical size for fracture-mediated debris formation. Experimental validation for other material systems remains an important direction for future work.

## Supporting Information

File 1 Visualizing sub-10 nm transfer films in metal–ceramic fretting using atomic force microscopy.

## Data Availability

All data that supports the findings of this study is available in the published article and/or the supporting information of this article.
