# Supplementary material for: Formation and compaction of sub-10 nm transfer films in metal–ceramic fretting
Source: Beilstein J Nanotechnol. 2026 Aug 28;17:1200–11. doi: 10.3762/bjnano.17.83 (PMC13550850; doi:10.3762/bjnano.17.83)
Supplement: Supplementary file 1 [file Beilstein_J_Nanotechnol-17-1200-s001.pdf]

BEILSTEIN JOURNAL OF NANOTECHNOLOGY

Supporting Information

for

Formation and compaction of sub-10 nm transfer films in metal–ceramic fretting

Tunç Çiftçi, Ozan Şahin, Thibault Roch and Bart Weber

Beilstein J. Nanotechnol. **2026,** *17,* 1200–1211. doi:10.3762/bjnano.17.83

Visualizing sub-10 nm transfer films in metal–ceramic fretting using atomic force microscopy

License and Terms: This is a supporting information file under the terms of the Creative Commons Attribution License (https://creativecommons.org/licenses/by/4.0). Please note that the reuse, redistribution and reproduction in particular requires that the author(s) and source are credited and that individual graphics may be subject to special legal provisions.
The license is subject to the *Beilstein Journal of Nanotechnology* terms and conditions: (https://www.beilstein-journals.org/bjnano/terms)

Hertzian Contact Calculations

To quantify the mechanical response at the sapphire–chromium interface under normal loading, we implemented a classical Hertzian contact model for a sphere-on-flat configuration. The goal was to compute the contact radius, peak contact pressure, and dimensionless ratios relevant for interpreting elastoplastic behavior. The model is based on the following assumptions:

- frictionless, elastic, isotropic contact between a spherical sapphire lens and a flat chromium-coated wafer,
- normal load $F = 0.15$ N, consistent with the experimental conditions,
- sphere radius $R = 1.6$ mm, matching the sapphire lens curvature.

Material Properties

The elastic properties were set as:

- sapphire: Young's modulus $E_1 = 335$ GPa, Poisson's ratio $\nu_1 = 0.25$ [1]
- chromium: Young's modulus $E_2 = 279$ GPa, Poisson's ratio $\nu_2 = 0.21$ [2,3].

The effective modulus E^* is computed using [4]:

$$\frac{1}{E^*} = \frac{1-\nu_1^2}{E_1} + \frac{1-\nu_2^2}{E_2} \quad \Rightarrow \quad E^* \approx 1.61 \times 10^{11} \text{ Pa} = 161 \text{ GPa}.$$

Hertzian Quantities

The following Hertzian parameters are computed [5]:

$$\begin{aligned}
a &= \left(\frac{3FR}{4E^*}\right)^{1/3} \approx 10.2\ \mu\text{m} \quad \text{(contact radius)},\\
A &= \pi a^2 \approx 3.3 \times 10^{-10}\ \text{m}^2 \quad \text{(contact area)},\\
p_{\text{max}} &= \frac{3F}{2\pi a^2} \approx 693 \text{ MPa} \quad \text{(maximum contact pressure)},\\
p_{\text{avg}} &= \frac{F}{\pi a^2} \approx 462 \text{ MPa} \quad \text{(average contact pressure)}.
\end{aligned}$$

Dimensionless Ratios and Plasticity Threshold

Here, a representative hardness of $H = 1$ GPa was assumed for the chromium film. To assess the tendency for plastic deformation, we calculated:

$$\frac{p_{\text{max}}}{E^*} \approx 0.0043 \quad \text{(normalized maximum contact pressure)},$$
$$\frac{p_{\text{avg}}}{E^*} \approx 0.003 \quad \text{(normalized average contact pressure)},$$
$$\frac{H}{E^*} \approx 0.0062 \quad \text{(hardness-to-modulus ratio)},$$
$$\frac{p}{H} \approx 0.5 - -0.7 \quad \text{(pressure-to-hardness ratio)}.$$

Interpretation

The calculated contact pressure approaches the predicted threshold for plastic deformation based on hardness comparisons, implying that yielding occurs within the contact zone. The values of p_0/E^* and p_0/H support a transition from elastic to elastoplastic deformation under the applied load, aligning with the simulation results discussed in the main text (Figure 2).

Elasto-plastic Contact Computation

We use an open-source implementation of a volume integral formulation of equilibrium for the solution of elastic–plastic rough contact [6,7] for solving the sapphire–chromium contact problem. We solve a coupled problem between (i) the elastic contact problem and (ii) the bulk elastic–plastic problem. First, (i) the elastic contact between the sapphire sphere and the chromium-coated wafer is modeled as a single rough surface in contact with a rigid flat surface, with the roughness from both surfaces mapped onto the single rough surface. The contact problem is solved with a compound roughness $h = h_1 - h_2$, with h_1 the roughness of the sapphire sphere and h_2 the one of the chromium-coated wafer measured with AFM. The surface topography size used for the computation is 34 × 34 μm^2 and is discretized into a 512 × 512 grid, with a pixel resolution of 66.4 × 66.4 nm. We use the effective mechanical properties of the sapphire–chromium contact as given previously, that

is, effective Young's modulus $E^* = 161 \pm 3\,\text{GPa}$. Solving this problem for a given contact load F_N provides the local pressures and displacements at the surface. The resulting pressures are used as the boundary conditions of (ii) the bulk elastic–plastic problem. The volume of the deformable material is discretized over a depth of 9.9 µm with 26 points. The bulk behavior is modeled with von Mises plasticity and isotropic hardening, with a yield stress $\sigma_y = 0.333GPa$ and a hardening modulus $E_h = E_2/100$. The plastic strains obtained with the bulk elastic–plastic problem are then used to modify the surface of the rough contact problem. The coupled problem is solved until the residual surface displacements has converged [7]. In Figure S1, we show the extent of the plastic strains just below the surface for the sapphire–chromium contact problem under a normal load $F_\text{N} = 150$ mN, emphasing the choice of an elasto-plastic computation.

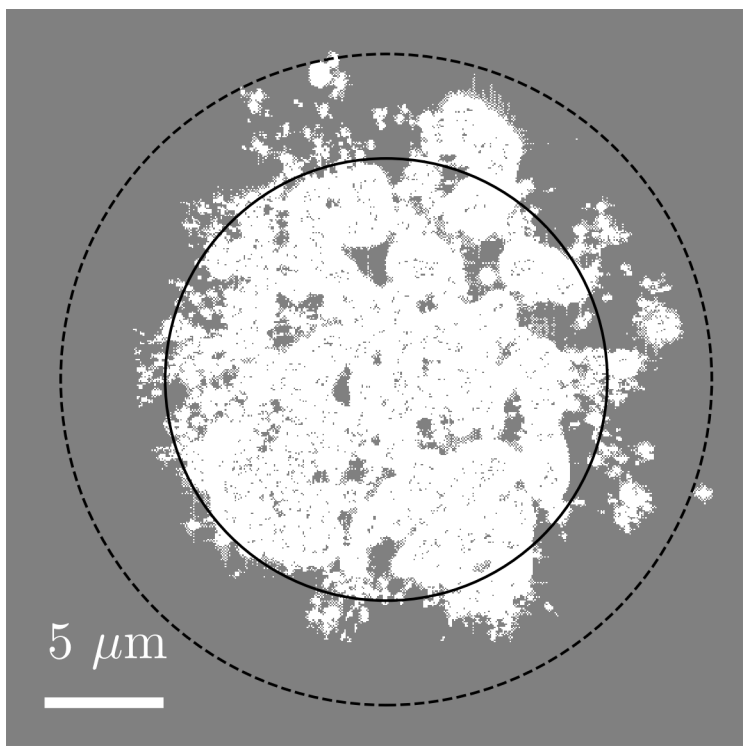

Figure S1: Map of the sub-surface plastic zones obtained from an elasto-plastic numerical simulation of the sapphire–chromium contact. The areas with plastic strains is spreading over the entire Hertzian contact radius (inner black circle).

Peripheral Ring Calculations

To escape from the contact, a debris particle must reach a region where the vertical gap between the sapphire lens and the wafer equals its height, as illustrated in Figure 7. Assuming a debris particle height of $z = 25$ nm and a sapphire lens radius of curvature $R = 1.59$ mm, we estimate the radial distance r_ring from the apex at which the gap equals z, accounting for the Hertzian indentation depth δ.

For a spherical surface of radius R, the geometric separation at a radial distance r is given by

$$d(r) = \frac{r^2}{2R}.$$

Solving for r yields

$$r_{\text{ring}} = \sqrt{2Rd}.$$

The Hertzian indentation depth can be approximated as

$$\delta = \frac{a^2}{2R},$$

where a is the Hertzian contact radius. Using $a = 10.2$ μm and $R = 1.59$ mm, we obtain

$$\delta = \frac{(10.2 \times 10^{-6})^2}{2(1.59 \times 10^{-3})} = \frac{104.04 \times 10^{-12}}{3.018 \times 10^{-3}} \approx 34.5 \times 10^{-9} \text{ m} = 34.5 \text{ nm}.$$

The total characteristic gap required for a particle of height z to escape is therefore

$$d = \delta + z = 34.5 \text{ nm} + 25 \text{ nm} = 59.5 \text{ nm}.$$

The corresponding radial distance from the apex is then

$$r_{\text{ring}} = \sqrt{2R(\delta + z)} = \sqrt{2(1.59 \times 10^{-3})(59.5 \times 10^{-9})} = \sqrt{1.796 \times 10^{-10}} \approx 1.34\times10^{-5} \text{ m} = 13.4 \text{ μm}.$$

This lies outside the Hertzian contact radius ($a = 10.2$ μm) by

$$r_{\text{ring}} - a \approx 13.4 - 10.2 \approx 3.2 \text{ μm}.$$

As the sapphire topography includes roughness peaks in reality, the chromium debris particles may be pushed outward by a larger distance than r_{ring}.

Results from Ball-2 from the same three-ball-puck

In the main Results section, we presented experimental outcomes from Ball 1 of the three-ball puck configuration (Figure 1c). Here, we present complementary results obtained from Ball 2, which was brought into contact with a different location on the same chromium-coated wafer. To further quantify the topographic changes and assess the spatial extent of material transfer, we employed a topographical difference method based on cross-sectional line profiles extracted from identical regions of AFM scans acquired before and after fretting. Figure S2a and Figure S2b show the AFM height maps of the sapphire apex before and after fretting, respectively.

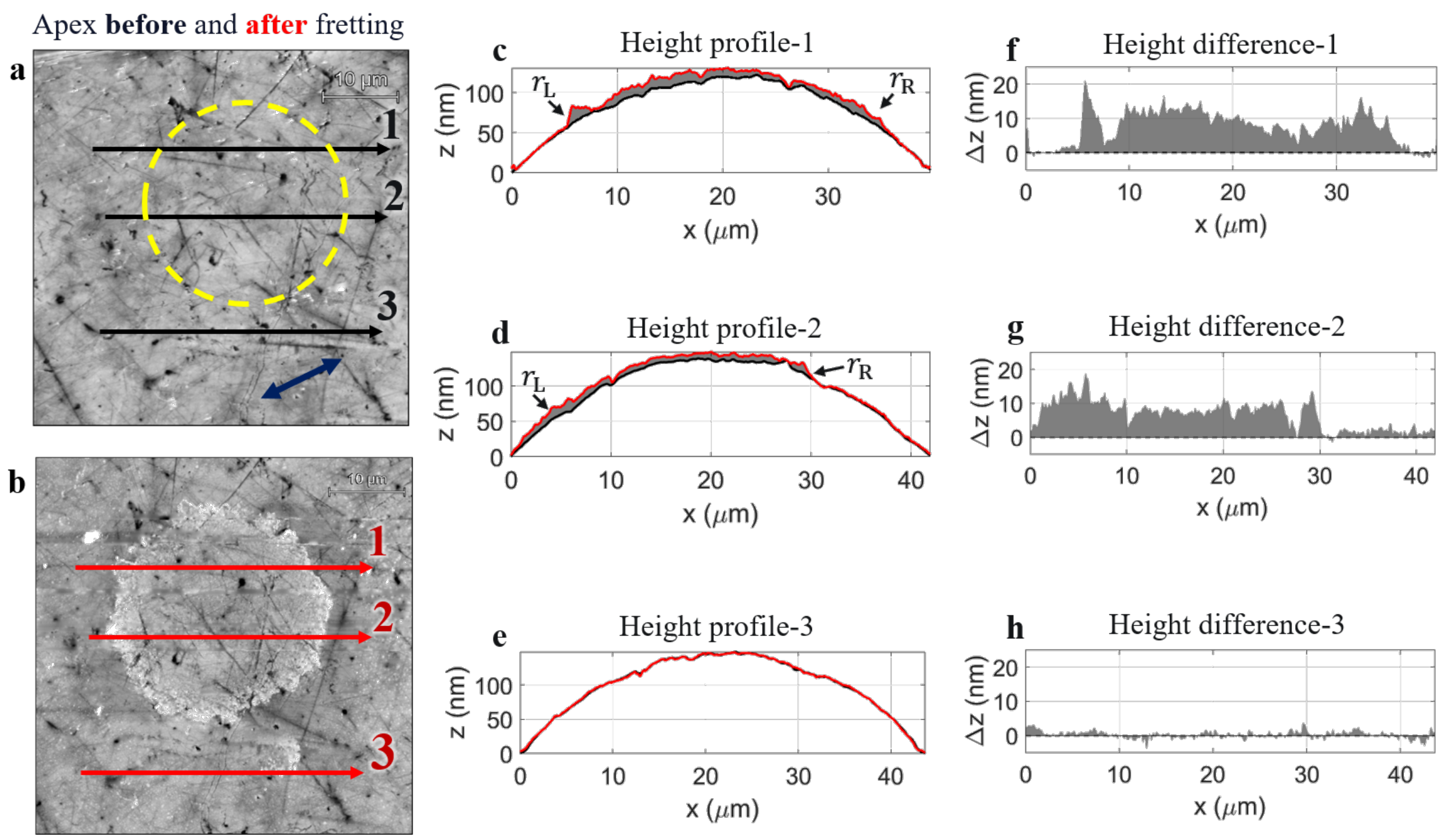

Figure S2: AFM height maps of the sapphire apex of Ball-2 (a) before and (b) after fretting (512 × 512 pixel). The yellow dashed circle indicates the Hertzian contact area, the dark blue arrow indicates the fretting direction. A thinner transfer film forms within the contact region in (b), consistent with the predicted Hertzian contact size, while the formed peripheral ring reveals a thicker profile as indicated by r_L and r_R. Trenches visible in both images are filled by transferred material. (c–e) Height profiles represent the black and red arrows from raw topographies before and after fretting (a an b). Each displayed height profile represents the average of 128 neighboring AFM scan lines, corresponding to a band width of approximately 8.5 µm. (f–h) Height difference plots taken from the gray colored areas between the black and the red profiles (c–e).

Height profiles extracted along the arrows in Figure S2a and Figure S2b quantitatively compare the topographic evolution in Figure S2c–h. Height and difference profiles shown in (Figure S2c,d) reveal a median height increase of approximately 5 nm within the Hertzian contact area (Figure S2f–g). Both profile 1 and profile 2 exhibit two peripheral "horns" in the difference profiles, denoted as r_L and r_R, corresponding to debris accumulation beyond the Hertzian contact area, with peak heights of 15–20 nm at the outer ring, located 7–9 µm outside the Hertzian zone. Furthermore, both profiles show that the broader grooves on the sapphire surface become progressively filled by transferred debris, as indicated by local height increases in the difference maps. Profile 3, taken outside the contact region, shows a nearly flat difference (~0.2 nm), confirming the stability and repeatability of the topographic measurement.

We also assessed complementary changes on the chromium-coated wafer, providing further evidence of interfacial material exchange and surface restructuring. Figure S3a,b shows corresponding AFM height maps of the chromium-coated wafer. After fretting, a deformed area within and around the Hertzian contact is clearly visible. The scar diameter and topography are consistent with the material transfer area observed on the sapphire (Figure S3b). Notably, the extended features within the wear scar suggest the possibility of a pre-slide phase before stable fretting motion was established, potentially due to slight nanometric tilt or bending of the wafer or stage system at the point of contact initiation.

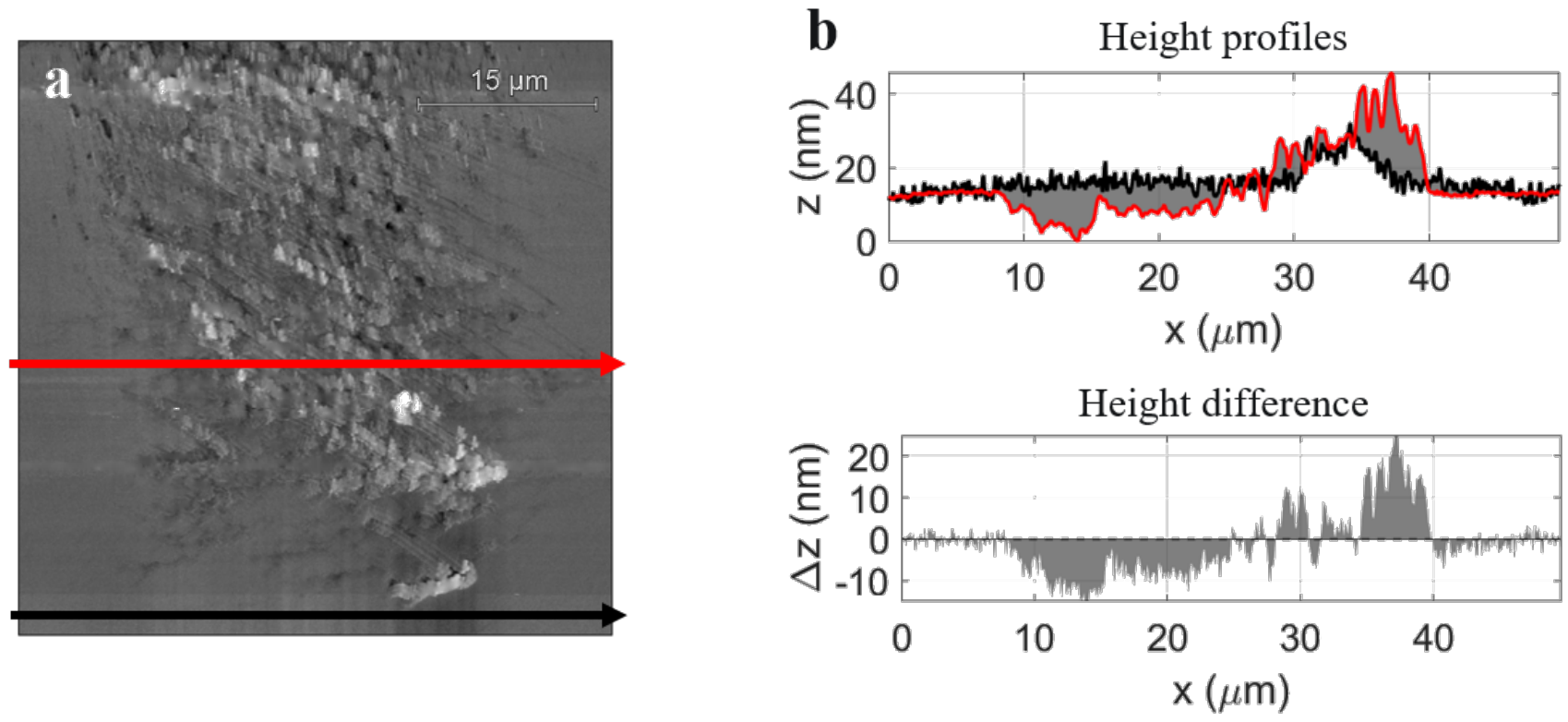

Figure S3: (a) AFM topograph of the chromium-coated wafer after fretting against Ball-2 (1024 × 1024 pixels). The post-fretting image shows a well-defined wear scar, which highlights local fretting-induced features. (b) Height profiles and the corresponding height-difference profile revealing the surface modulation associated with fretting. Each displayed height profile represents the average of 128 neighboring AFM scan lines, corresponding to a band width of approximately 8.5 µm.

Last, the adhesion mapping results shown in Figure S4 complement the height data and reveal spatially resolved contrast. On the sapphire apex (Figure S4a), the transferred chromium region exhibits significantly lower adhesion compared to the pristine sapphire areas outside the wear scar, indicating a distinct surface composition within the transfer film. On the chromium-coated wafer (Figure S4b), the wear scar displays a higher adhesion signal relative to the surrounding smooth chromium surface. Importantly, the same AFM tip was used for all adhesion measurements, both before and after fretting. The observed adhesion contrast between the sapphire and chromium surfaces confirms complementary interfacial evolution at both sides of the contact.

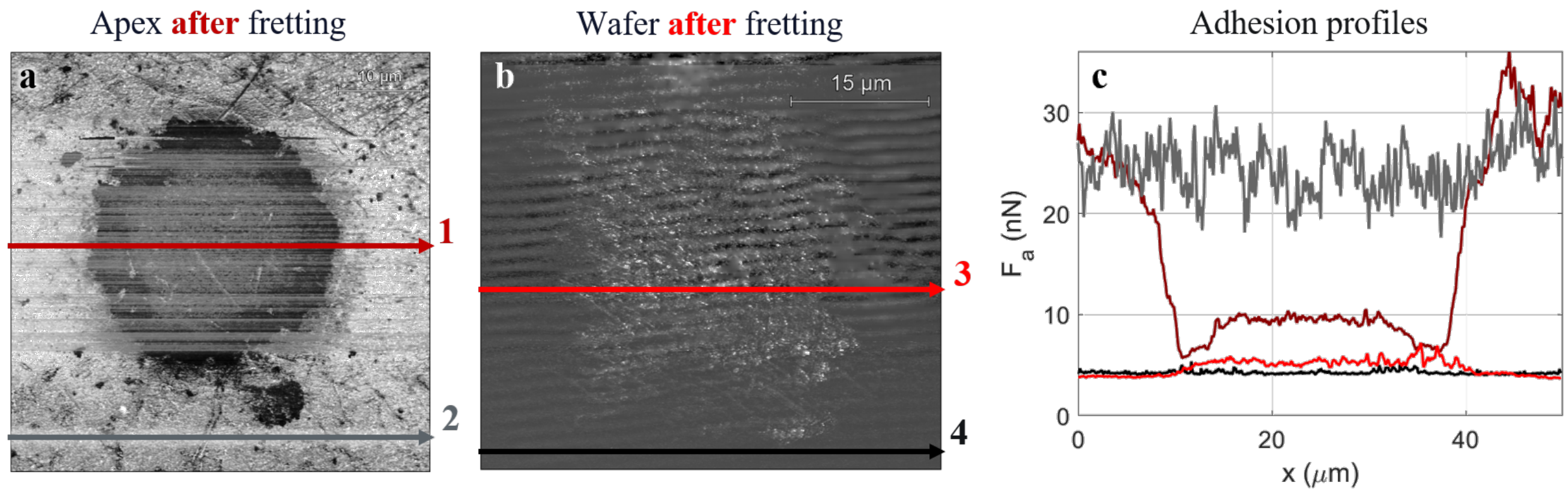

Figure S4: Adhesion maps of (a) the sapphire apex of Ball-2 and (b) the wear scar on the chromium surface after fretting measured in PeakForce QNM mode using the same AFM tip (Adama Innovations, AD-2.8-AS). (c) Adhesion profiles extracted along the corresponding marked positions (in a and b), represent the average of 128 neighboring scan lines. The dark red profile along the transferred material at the ball apex exhibits lower adhesion compared to the dark gray profile from surrounding pristine sapphire regions. The red profile along the wear scar on the chromium-coated wafer shows higher adhesion compared to the black profile from the surrounding flat chromium film. The contrast between reduced adhesion on the sapphire and enhanced adhesion on the chromium confirms asymmetric interfacial evolution during material transfer, correlating with the topographic profiles shown in Figure S2 and Figure S3.

Additional results

Figure S5 and Figure S6 show the results of an independent repeat of the fretting experiment reported in the main text. Overall, the results are similar. Variation in transfer film and peripheral ring thickness is likely sensitive to the age and cleaning history of the chromium sample. This will be investigated in more detail in future work.

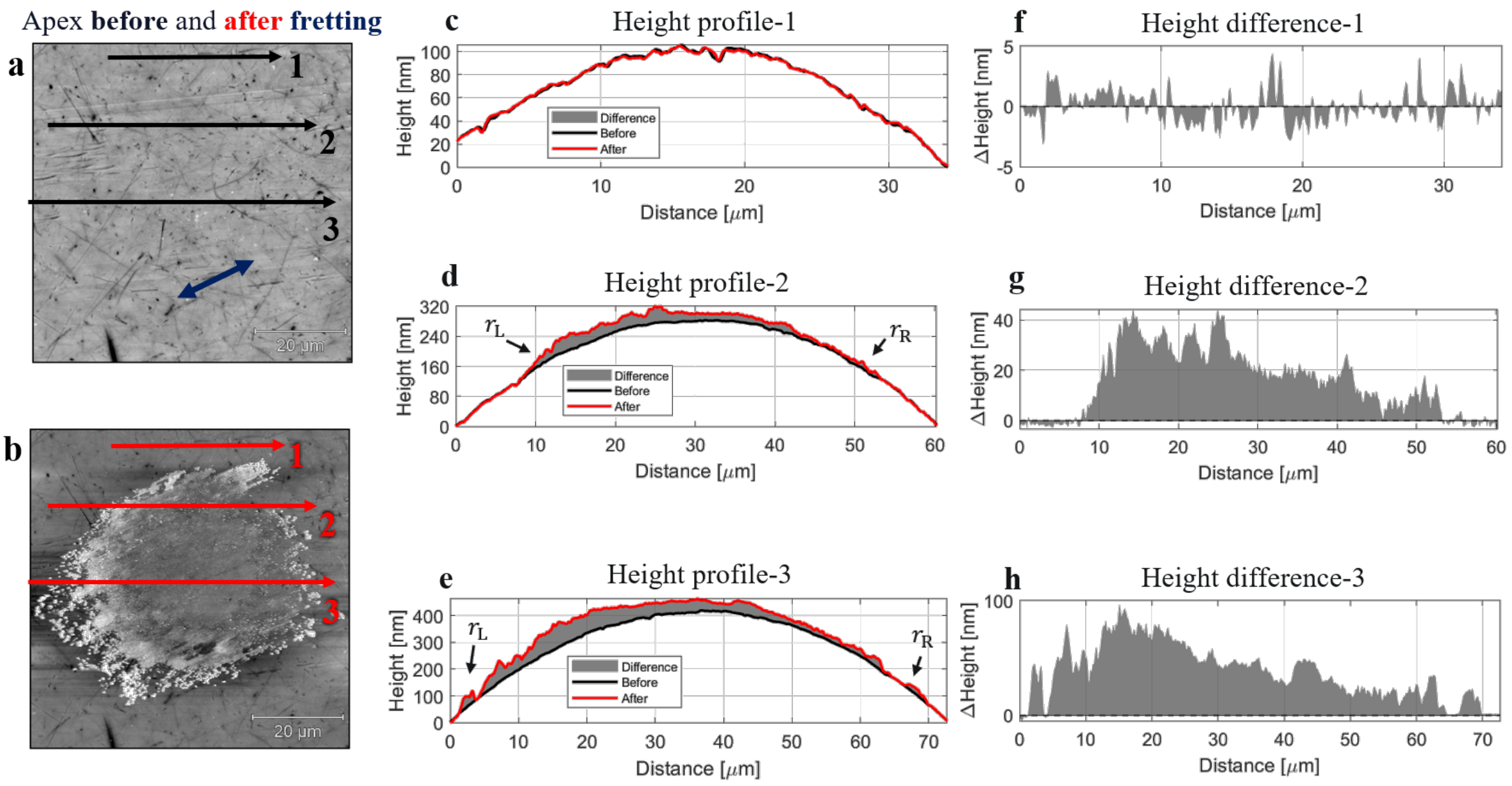

Figure S5: AFM height maps of the sapphire apex of Ball-1 (from the repeat experiment) (a) before and (b) after fretting (512 × 512 pixel). A thinner transfer film forms within the contact region in (b), consistent with the predicted Hertzian contact size, while the formed peripheral ring reveals a thicker profile as indicated by r_L and r_R. Trenches visible in both images are filled by transferred material. (c–e) Height profiles represent the black and red arrows from raw topographies before and after fretting (a an b). Each displayed height profile represents the average of 128 neighboring AFM scan lines, corresponding to a band width of approximately 8.5 μm. Height difference plots (right) taken from the gray colored areas between the black and the red profiles (c–e).

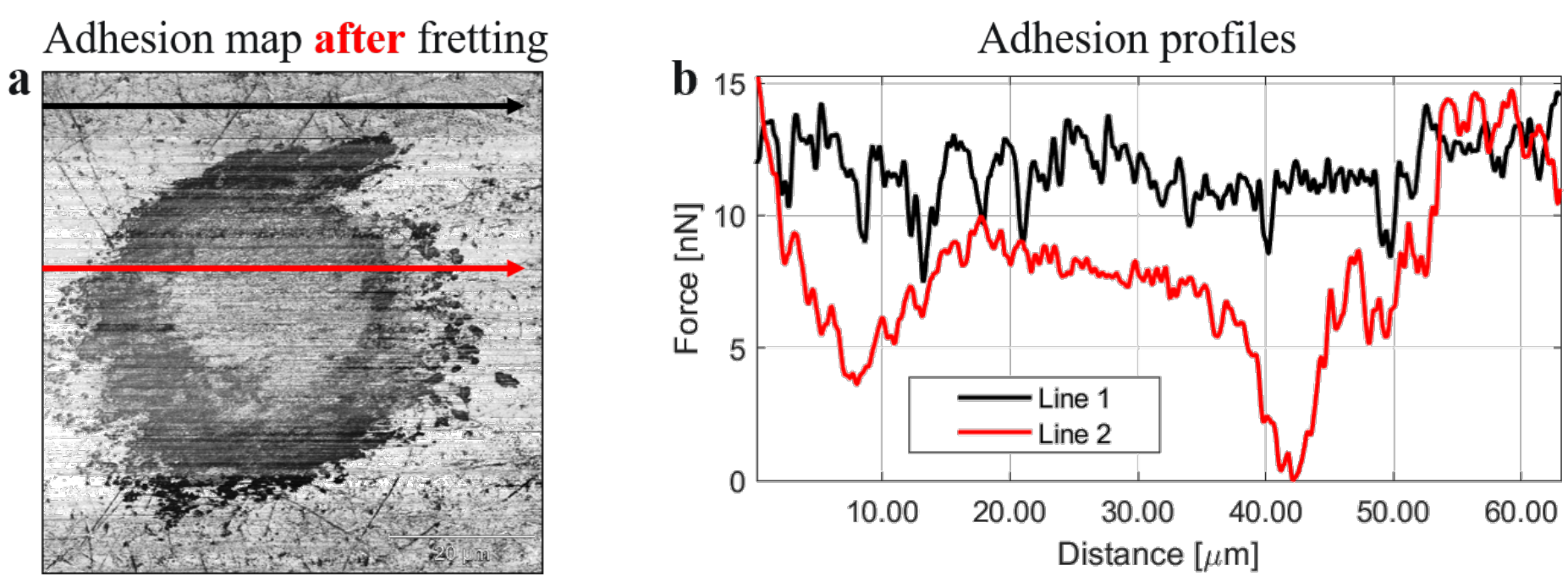

Figure S6: Adhesion maps of (a) the sapphire apex of Ball-1 after fretting measured in PeakForce QNM mode using the same AFM tip (Adama Innovations, AD-2.8-AS). (b) Adhesion profiles extracted along the corresponding marked positions (in a), represent the average of 128 neighboring scan lines. The red profile along the transferred material at the ball apex exhibits lower adhesion compared to the black profile from surrounding pristine sapphire regions. The contrast in adhesion correlates with the topographic profiles shown in Figure S5.

References

1. Properties of Sapphire. https://www.shinkosha.com/english/wp-content/uploads/sites/2/2021/07/properties-of-sapphire_eng.pdf (accessed 2026-08-25).

2. Alakiozidis, I.; Hunt, C.; Smith, A. D.; Maric, M.; Shah, Z.; Ambard, A.; Frankel, P. *J. Nucl. Mater.* **2025**, *604*, 155492. doi:10.1016/j.jnucmat.2024.155492.

3. WebElements Ltd., Chromium: Physical Properties. https://www.webelements.com/chromium/physics.html (accessed 2026-08-25).

4. Chaudhry, V.; Kailas, S. V. *Wear* **2013**, *301*, 524–539. doi:10.1016/j.wear.2013.01.084.

5. Johnson, K. L. *Contact Mechanics*; Cambridge University Press: Cambridge, UK, 1985.

6. Frerot, L.; Anciaux, G.; Rey, V.; Pham-Ba, S.; Molinari, J.-F. *J. Open Source Software* **2020**, *5*, 2121. doi:10.21105/joss.02121.

7. Frerot, L.; Bonnet, M.; Molinari, J.-F.; Anciaux, G. *Comput. Methods Appl. Mech. Eng.* **2019**, *351*, 951–976. doi:10.1016/j.cma.2019.04.006.
